# Non-locality Correlation in Two Driven Qubits Inside an Open Coherent Cavity: Trace Norm Distance and Maximum Bell Function

**DOI:** 10.1038/s41598-019-55548-2

**Published:** 2019-12-23

**Authors:** A. -B. A. Mohamed, H. Eleuch, C. H. Raymond Ooi

**Affiliations:** 1 Department of Mathematics, College of Science and Humanities in Al-Aflaj, Prince Sattam bin Abdulaziz University, Al-Aflaj, Saudi Arabia; 2 Faculty of Science, Assiut University, Assiut, Egypt; 3 Department of Applied Sciences and Mathematics, College of Arts and Sciences, Abu Dhabi University, Abu Dhabi, UAE; 4 Institute for Quantum Science and Engineering, Texas A&M University, College Station, Texas 77843 USA; 5 Department of Physics, University of Malaya, 50603 Kuala Lumpur, Malaysia

**Keywords:** Quantum information, Single photons and quantum effects

## Abstract

We analytically investigate two separated qubits inside an open cavity field. The cavity is initially prepared in a superposition coherent state. The non-locality correlations [including trace norm measurement induced non-locality, maximal Bell-correlation, and concurrence entanglement] of the two qubits are explored. It is shown that, the generated non-locality correlations crucially depend on the decay and the initial coherence intensity of the cavity field. The enhancement of the initial coherence intensity and its superposition leads to increasing the generated non-locality correlations. The phenomena of sudden birth and death entanglement are found.

## Introduction

Two-level system (qubit) is not only the key element in various fields of the modern physics, such as quantum optics and collision physics^1,2^, but also the fundamental building block of modern applications ranging from quantum control^3^ to quantum processing^4^.

Due to the rapid development of experiments in macroscopic solid state physics, the artificial two-level atoms qubits based on the superconducting (SC) circuits^5,6^ and quantum dots (QDs)^7^ have been recognized as possible candidate for quantum processing. The SC-qubits have macroscopic quantum coherence. It may be helpful for the realization of the conditional two-qubit gate and quantum hybrid system^8,9^. Embedding QD-qubits in microcavities enhances the light extraction efficiency via the Purcell effect and permits the study of cavity QED effects in solid-state systems^10–12^. Experimentally, the qubit-photon interaction was intensively investigated^8,9,13–16^.

The dissipation eradicates the useful quantum coherence and correlations^17,18^. The existence of dissipative qubits, such as in amorphous solids, is a longstanding problem in solid-state physics^19,20^.

There is a growing interest in the dynamics of non-local correlations (NLCs) beyond the quantum entanglement (QE)^21^, which is a unique type that has a major role in quantum processing^22^. However, QE does not have all of the non-classical properties of the quantum correlations^23^. While NLCs between the parts of a system in a pure state is fully characterized by their entanglement, mixed states may possess NLCs even if they are not entangled. New types of NLCs were introduced beyond QE^24^ as: measurement-induced disturbance^25^, quantum discord^26^, and that was determined by using *p*-norms such as; the Hilbert-Schmidt norm, Schatten one-norm and Bures norm. Due to analytic difficulty to the quantum discord, the geometric correlations appeared via geometric quantum discord (GQD) and the measurement-induced nonlocality (MIN)^27^ were proposed by using the 2-norm^28^. However, these measurements that are based on 2-norm have been proved to be incompetent measures of NLC^29^. Consequently, the GQD and MIN are derived using 1-norm (trace norm)^30–32^. Moreover, non-classical correlations registered by Bell inequality violation^33^ (that constitutes one of the most striking phenomena ever observed in nature) is used as an indicator of non-local quantum properties.

While NLCs between the parts of a system in a pure state is fully characterized by their entanglement, mixed states may possess NLCs even if they are not entangled. Unlike entanglement, quantum discord is rather robust against decoherence^34^. It is shown that the dynamics of GQD is more robust than the thermal entanglement. Also, the measures of MINs can be considered as one type of quantum correlation that differs from entanglement and quantum discord^30,31,35^.

Despite the complexity of the suggested model, it is significant to introduce an analytical description of two optically driven qubits inside an open cavity, that is initially prepared in a coherent state. Therefore, the non-locality correlations [including, trace norm measurement induced non-locality, maximal Bell-correlation] and the entanglement via the concurrence could be determined.

In Sec. 2, the model of (two-qubit)-cavity system and its analytical solution are introduced. In Sec. 3, the non-locality correlation functions is displayed. We discuss the results of the non-locality correlations in Sec. 4. Finally, we conclude in Sec. 5.

## The Physical Model

The studied system is constituted by two sufficiently separated identical artificial two-level atoms (considered as two qubits *A* and *B*) resonantly interacting with an open cavity. The two atoms are separated by a distance much larger than their size, consequently their dipole-dipole interplay can be neglected^36^. In the rotating wave approximation, the total Hamiltonian is:

1 $$\begin{array}{rcc}\hat{H} & = & {\omega }_{0}{\hat{a}}^{\dagger }\hat{a}+{\omega }_{0}\sum _{i=A,B}{\hat{\sigma }}_{i}^{z}+\sum _{i=A,B}({\lambda }_{i}\hat{a}{\hat{\sigma }}_{i}^{-}+{\lambda }_{i}^{\ast }{\hat{a}}^{\dagger }{\hat{\sigma }}_{i}^{+}),\end{array}$$

where *ω*_0_ represent the qubits and the cavity frequency. ${\hat{a}}^{\dagger }$ and $\hat{a}$ are respectively the creation and annihilation operators of the cavity mode. The operators ${\hat{\sigma }}_{i}^{\pm }$ and ${\hat{\sigma }}_{i}^{z}$ are the Pauli matrices which are defined by the upper states |1〉_*i*_, and lower states |0〉_*i*_. *λ*_*i*_ designate the coupling between the cavity and the qubits. Here, we focus on the case where *λ*_*i*_ = *λ*.

If we consider only the dissipative term of the dipole decay of the qubits, the dynamic of the system is given by^37^

2 $$\begin{array}{rcl}\frac{\partial \hat{\rho }(t)}{\partial t} & = & -i[\hat{H},\hat{\rho }(t)]\\  &  & +\sum _{i=A,B}{\gamma }_{i}([{\hat{\sigma }}_{i}^{-},\hat{\rho }(t){\hat{\sigma }}_{i}^{+}]+[{\hat{\sigma }}_{i}^{-}\hat{\rho }(t),{\hat{\sigma }}_{i}^{+}]),\end{array}$$

where *γ*_*i*_ are spontaneous emission rates of the two qubits, which are treated by coupling each qubit to reservoir. In the basis states {|1〉 = |1_*A*_1_*B*_, *n*〉, |2〉 = |1_*A*_0_*B*_, *n* + 1〉, |3〉 = |0_*A*_1_*B*_, *n*_+1_〉, |4〉 = |0_*A*_0_*B*_, *n* + 2〉}, the dressed states, |Ψ_*i*_^*m*^〉(*i* = 1–4), are

3 $$\,(\begin{array}{c}|{\varPsi }_{1}^{n}\rangle \\ |{\varPsi }_{2}^{n}\rangle \\ |{\varPsi }_{3}^{n}\rangle \\ |{\varPsi }_{4}^{n}\rangle \end{array})=A(\begin{array}{c}\mathrm{|1}\rangle \\ \mathrm{|2}\rangle \\ \mathrm{|3}\rangle \\ \mathrm{|4}\rangle \end{array}),A=(\begin{array}{cccc}{\tilde{a}}_{n} & 0 & 0 & -{\tilde{b}}_{n}\\ 0 & \frac{1}{\sqrt{2}} & -\frac{1}{\sqrt{2}} & 0\\ {b}_{n} & -\frac{1}{2} & -\frac{1}{2} & {a}_{n}\\ {b}_{n} & \frac{1}{2} & \frac{1}{2} & {a}_{n}\end{array})\mathrm{}.$$

where ${a}_{n}=\sqrt{\frac{n+2}{2(2n+3)}},\,{b}_{n}=\sqrt{\frac{n+1}{2(2n+3)}}$ and $\tilde{a}{(\tilde{b})}_{n}=a{(b)}_{n}\sqrt{2}$. In the case of high −*Q* cavity (${\gamma }_{i}\ll {\lambda }_{i}$), we apply the dressed-states representation (DSR) based on the Hamiltonian eigenstates^38–40^. The operators of the qubits, |1〉_*ii*_〈0|, of Eq. (7) are written in the DSR.

In this paper we focus on the case where the two qubits are initially in the excited state (uncorrelated state), i.e., ${\hat{\rho }}_{AB}(0)$ = |1_*A*_1_*B*_〉〈1_*A*_1_*B*_|. While the wave function of cavity mode field is initially prepared in the superposition coherent state: |*α*〉 + *κ*|−*α*〉, where *α* is the intensity of the coherent state, i.e.,

4 $$\begin{array}{rcl}\,{\hat{\rho }}_{F}(0) & = & \sum _{m,n=0}{Q}_{m,n}|m\rangle \langle n|,\\  & = & \frac{[1+\kappa {(-1)}^{m}][1+\kappa {(-1)}^{n}]}{[1+{\kappa }^{2}+2\kappa {e}^{-2N}]\sqrt{m!n!}}{N}^{\frac{m+n}{2}}{e}^{-N}|m\rangle \langle n|.\end{array}$$

*N* = |*α*|^2^ designs the mean photon number and *κ* = 0 and 1 are taken respectively for the coherent state and the superposition coherent state. Coherent states and their superpositions are proposed as major elements for the realization of quantum processing. Using the dressed states space, {|Ψ_*i*_^*m*^〉}, of Eq. (3), the initial total density matrix in DSR is rewritten as:

5 $$\begin{array}{ccc}\,W(0) & = & \sum _{m,n=0}2{f}_{aa}|{\Psi }_{1}^{m}\rangle \langle {\Psi }_{1}^{n}|+{f}_{ba}^{mn}\sqrt{2}[{\hat{h}}_{31}^{mn}+{\hat{h}}_{41}^{mn}]\\  &  & +{f}_{bb}^{mn}[{\hat{h}}_{33}^{mn}+{\hat{h}}_{34}^{mn}+{\hat{h}}_{43}^{mn}+{\hat{h}}_{44}^{mn}]+{f}_{ab}^{mn}\sqrt{2}[{\hat{h}}_{13}^{mn}|+{\hat{h}}_{14}^{mn}],\end{array}$$

where *f*_*rs*_^*mn*^ = *Q*_*m*,*n*_*r*_*m*_*s*_*n*_(*r*, *s* = *a*, *b*), ${\hat{h}}_{kl}^{mn}$ = |Ψ_*k*_^*n*^〉〈Ψ_*l*_^*n*^|.

In the basis states of the two qubits {|1〉 = |1_*A*_1_*B*_, *n*〉, |2〉 = |1_*A*_0_*B*_, *n* + 1〉, |3〉 = |0_*A*_1_*B*_, *n* + 1〉, |4〉 = |0_*A*_0_*B*_, *n* + 2〉}, Eq. 2 becomes

6 $$\hat{\rho }(t)=\mathop{\sum }\limits_{ij=0}^{4}\mathop{\sum }\limits_{mn=0}^{\infty }{\langle i|{e}^{-i\hat{H}t}W(t){e}^{i\hat{H}t}|j\rangle }_{mn}(t)\,|i\rangle \langle j|\mathrm{}.$$

To calculate ${\langle i|{e}^{-i\hat{H}t}W(t){e}^{i\hat{H}t}|j\rangle }_{mn}(t)$, we used the following canonical transform; $W(t)={e}^{i\hat{H}t}\hat{\rho }(t){e}^{-i\hat{H}t}$ in Eq. (7) to became:

7 $$\dot{W}(t)=\sum _{i=A,B}{\gamma }_{i}{e}^{i\hat{H}t}([{\hat{\sigma }}_{i}^{-},\hat{\rho }(t){\hat{\sigma }}_{i}^{+}]+[{\hat{\sigma }}_{i}^{-}\hat{\rho }(t),{\hat{\sigma }}_{i}^{+}]){e}^{-i\hat{H}t}\mathrm{}.$$

If *i* ≠ *j*, the elements 〈Ψ_*k*_^*i*^|*W*(*t*)|Ψ_*l*_^*j*^〉 are calculated from

8 $$\langle {\Psi }_{k}^{i}|W(t)|{\Psi }_{l}^{j}\rangle ={e}^{-({\gamma }_{A}+{\gamma }_{B}){\beta }_{kl}t}\langle {\Psi }_{k}^{i}|W\mathrm{(0)|}{\Psi }_{l}^{j}\rangle ,$$

with ${\beta }_{12}^{ij}={\beta }_{21}^{ji}=(2{a}_{i}^{2}+\frac{1}{2})$, ${\beta }_{23}^{ij}={\beta }_{32}^{ji}={\beta }_{24}^{ij}={\beta }_{42}^{ji}=({b}_{j}^{2}+\frac{3}{4})$, *β*_22_^*ij*^ = 1, ${\beta }_{13}^{ij}={\beta }_{31}^{ji}={\beta }_{41}^{ij}={\beta }_{14}^{ji}=$ $(2{a}_{i}^{2}+{b}_{j}^{2}+\frac{1}{4})$ and ${\beta }_{33}^{ij}={\beta }_{34}^{ij}={\beta }_{43}^{ij}={\beta }_{44}^{ij}=({b}_{i}^{2}+{b}_{j}^{2}+\frac{1}{2})$.

While, if *i* = *j*, the elements 〈Ψ_*k*_^*i*^|*W*(*t*)|Ψ_*l*_^*i*^〉 are calculated from

9 $$\begin{array}{rcl}{\dot{A}}_{i} & = & 2\gamma {b}_{i}^{2}{B}_{i+1}+\gamma {b}_{i}^{2}{C}_{i+1}-4\gamma {a}_{i}^{2}\,{A}_{i},\\ {\dot{B}}_{i} & = & 2\gamma {a}_{i+1}^{2}{A}_{i+1}+\gamma {b}_{i+1}^{2}{C}_{i+1}-\gamma {B}_{i},\\ {\dot{C}}_{i} & = & 2\gamma {a}_{i+1}^{2}{A}_{i+1}+2\gamma {a}_{i}^{2}{B}_{i+1}+2\gamma {\chi }_{i+1}{C}_{i+1}-\gamma {\varphi }_{i}{C}_{i}.\end{array}$$

where *γ* = *γ*_*A*_ + *γ*_*B*_, *A*_*i*_ = 〈Ψ_1_^*i*^|*W*(*t*)|Ψ_1_^*i*^〉, *B*_*i*_ = 〈Ψ_2_^*i*^|*W*(*t*)|Ψ_2_^*i*^〉, *C*_*i*_ = 〈Ψ_3_^*i*^|*W*(*t*)|Ψ_3_^*i*^〉 + 〈Ψ_4_^*i*^|*W*(*t*)|Ψ_4_^*i*^〉, ${\chi }_{i+1}=\frac{1}{2}({a}_{i}^{2}+{b}_{i+1}^{2})$ and ${\varphi }_{i}=2{b}_{i}^{2}+\frac{1}{2}$. The Eq. (9) is exactly solvable for the case where each state has at most *N* photons only, i.e, *A*_*N*+1_ = *B*_*N*+1_ = *C*_*N*+1_ = 0, the case *N* → ∞ could be considered^38^. Equation (9) gives at *i* = *N*,

10 $$\begin{array}{rcl}{A}_{i}(t) & = & {e}^{-4\gamma {a}_{i}^{2}t}[{A}_{i}\mathrm{(0)}+\gamma {b}_{i}^{2}{\int }_{0}^{t}[2{B}_{i+1}+{C}_{i+1}]{e}^{4\gamma {a}_{i}^{2}t}dt],\\ {B}_{i}(t) & = & {e}^{-\gamma t}[{B}_{i}\mathrm{(0)}+\,\gamma {\int }_{0}^{t}\,[2{a}_{i+1}^{2}{A}_{i+1}+{b}_{i+1}^{2}{C}_{i+1}]{e}^{\gamma t}dt],\\ {C}_{i}(t) & = & {e}^{-\gamma {\varphi }_{i}t}[{C}_{i}\mathrm{(0)}+2\gamma {\int }_{0}^{t}[{a}_{i+1}^{2}{A}_{i+1}+{a}_{i}^{2}{B}_{i+1}+{\chi }_{i+1}{C}_{i+1}]{e}^{\gamma {\varphi }_{i}t}dt].\end{array}$$

Therefore ${A}_{N}(t)={e}^{-4\gamma {a}_{i}^{2}t}{A}_{N}(0),\,{B}_{N}(t)={e}^{-\gamma t}{B}_{N}(0),\,{C}_{N}(t)={e}^{-\gamma {\varphi }_{N}t}{C}_{N}(0)$. at *i* = *N* − 1, we get

$$\begin{array}{rcl}{A}_{N-1}(t) & = & {e}^{-4\gamma {a}_{N-1}^{2}t}[{A}_{N-1}\mathrm{(0)}\\  &  & +\gamma {b}_{N-1}^{2}{\int }_{0}^{t}\mathrm{[2}{B}_{N}+{C}_{N}]{e}^{4\gamma {a}_{N-1}^{2}t}dt],\\ {B}_{N-1}(t) & = & {e}^{-\gamma t}[{B}_{N-1}\mathrm{(0)}+\gamma {\int }_{0}^{t}\mathrm{[2}{a}_{N}^{2}{A}_{N}+{b}_{N}^{2}{C}_{N}]{e}^{\gamma t}dt],\\ {C}_{N-1}(t) & = & {e}^{-\gamma {\varphi }_{N-1}t}[{C}_{N-1}\mathrm{(0)}+2\gamma {\int }_{0}^{t}[{a}_{N}^{2}{A}_{N}+{a}_{i}^{2}{B}_{N}+{\chi }_{N}{C}_{N}]{e}^{\gamma {\varphi }_{N-1}t}dt].\end{array}$$

Then we can calculate for *i* = *N* − 2, *N* − 3, ..., 0. The density operator of the qubits *ρ*^*AB*^(*t*) can be determined, by tracing the cavity field degrees of freedom as:

11 $${\rho }^{AB}(t)={{\rm{tr}}}_{F}\{\rho (t)\}=\mathop{\sum }\limits_{k\mathrm{=0}}^{\infty }\langle k|\rho (t)|k\rangle \mathrm{}.$$

Now, we can explore the time evolution of the non-local correlations.

## Non-Locality and Concurrence Quantifiers

We adopt as non-locality quantifiers: the trace norm measurement induced non-locality (MIN) and the Bell function. These measures will be compared to the concurrence as a quantifier of entanglement.

### Concurrence

The concurrence^41^ is one of the most used measures of the entanglement between two qubits. It is defined as,

12 $$C(t)=\,{\rm{\max }}\{0,\sqrt{{\lambda }_{1}}-\mathop{\sum }\limits_{i=2}^{4}\,\sqrt{{\lambda }_{i}}\};\,{\lambda }_{i} > {\lambda }_{i+1},$$

where *λ*_*i*_ are the eigenvalues of the following matrix:

$$T={\rho }^{AB}(t)({\sigma }_{y}\otimes {\sigma }_{y}){\rho }^{\ast AB}(t)({\sigma }_{y}\otimes {\sigma }_{y}).$$

### Trace-norm MIN

Firstly, the measurement induced non-locality for a two-qubit state *ρ*^*AB*^(*t*) is defined via Hilbert-Schmidt norm (2-norm)^27^. Unfortunately, just like geometric quantum discord based on 2-norm, it may change under trivial local reversible operations on an unmeasured subsystem of *ρ*^*AB*^(*t*)^29^. To address this issue, the MIN based on trace norm (1-MIN) and others have been introduced by^30–32^. The modified versions of the *p*-MIN based on the Schatten *p*-norm is given by^30,31^

13 $${M}_{p}({\rho }^{AB}(t))=\mathop{{\rm{\max }}}\limits_{\chi \in {\varPi }^{A}}{\Vert {\rho }^{AB}(t)-{\Pi }^{A}({\rho }^{AB}(t))\Vert }_{p}^{p}.$$

where ${\Vert A\Vert }_{p}$ is the Schatten *p*-norm of a matrix/vector *A*. Here we use 1-MIN that represents the maximal trace distance between the pre-measurement state and the post-measurement state caused by the locally invariant measurements. the trace-norm MIN in explicit form can be written as:

14 $$M(t)=\{\begin{array}{cc}\frac{\sqrt{{\chi }_{+}}+\sqrt{{\chi }_{-}}}{2{\Vert \overrightarrow{{\rm{x}}}\Vert }_{1}}, & {\rm{If}}\,\overrightarrow{{\rm{x}}}\ne \mathrm{0;}\\ 2\,{\rm{\max }}\,\{|{r}_{11}|,|{r}_{22}|,|{r}_{33}|\}, & {\rm{If}}\,\overrightarrow{{\rm{x}}}=0.\end{array}$$

where ${\chi }_{\pm }=\alpha \pm 2\sqrt{\beta }{\Vert \overrightarrow{{\rm{x}}}\Vert }_{1}$, $\alpha ={\Vert C\Vert }_{2}^{2}{\Vert x\Vert }_{1}^{2}-{\sum }_{i}\,{r}_{ii}^{2}{x}_{i}^{2}$, *C* = [*r*_*ii*_] and $\beta ={\sum }_{\langle ijk\rangle }{x}_{i}^{2}{r}_{jj}^{2}{r}_{kk}^{2}$, the summation of *β* runs over all the cyclic permutations of 1,2,3. where where *x*_*i*_ = *Tr*(*ρ*^*AB*^*(t*)(*σ*_*i*_ ⊗ *I*)) are the elements of the local Bloch vector *x*, while, *r*_*mn*_ = *tr*{*ρ*^*AB*^*(t*)(*σ*_*m*_ ⊗ *σ*_*n*_)} represent the components of the correlation matrix *R* = [*r*_*mn*_]^28^. $\overrightarrow{\sigma }=({\sigma }_{1},{\sigma }_{2},{\sigma }_{3})$ represents a vector of the Pauli spin matrices.

If the elements of density matrix *ρ*^*AB*^(*t*) are denoted by: *z*_*ij*_ = 〈*i*|*ρ*^*AB*^(*t*)|*j*〉 = *e*_*ij*_ + *id*_*ij*_, then

15 $$\overrightarrow{{\rm{x}}}={({e}_{13}+{e}_{24},{d}_{31}+{d}_{42},{z}_{11}+{z}_{22}-1/2)}^{t},$$

while, the correlation matrix *R* for a general bipartite quantum state *ρ*^*AB*^(*t*) is given by

16 $${\bf{R}}=(\begin{array}{ccc}{e}_{23}+{e}_{14} & {d}_{23}-{d}_{14} & {e}_{13}-{e}_{24}\\ {d}_{41}-{d}_{23} & {e}_{23}-{e}_{14} & {d}_{13}+{d}_{24}\\ {e}_{12}-{e}_{34} & {d}_{34}-{d}_{12} & {z}_{11}+{z}_{44}-\frac{1}{2}\end{array})\mathrm{}.$$

**(ii) - Maximum Bell function:**

The maximal value of the Bell function *B*_*max*_(*t*), is considered as an indicator of non-locality correlation^33^. If *B*_max_(*t*) > 2, then the Bell’s inequality is violated, i.e., *B*_max_(*t*) locates the nonlocal quantum correlations when its value is above 2 (the classical threshold). Here, we use the Bell function defined by

17 $$B(t)=2\sqrt{{S}_{{\rm{\max }}}}-1,$$

where, *S*_max_ is the summation of the two largest eigenvalues for the matrix $U={{\rm{R}}}^{\dagger }{\rm{R}}$, R represents the correlation matrix of a two-qubit state *ρ*^*AB*^. The function *B*(*t*) identifies NLC when it is above the classical threshold 1.

## NLC Dynamics

### Effect of the coherent cavity superposition

**Figure 1:**
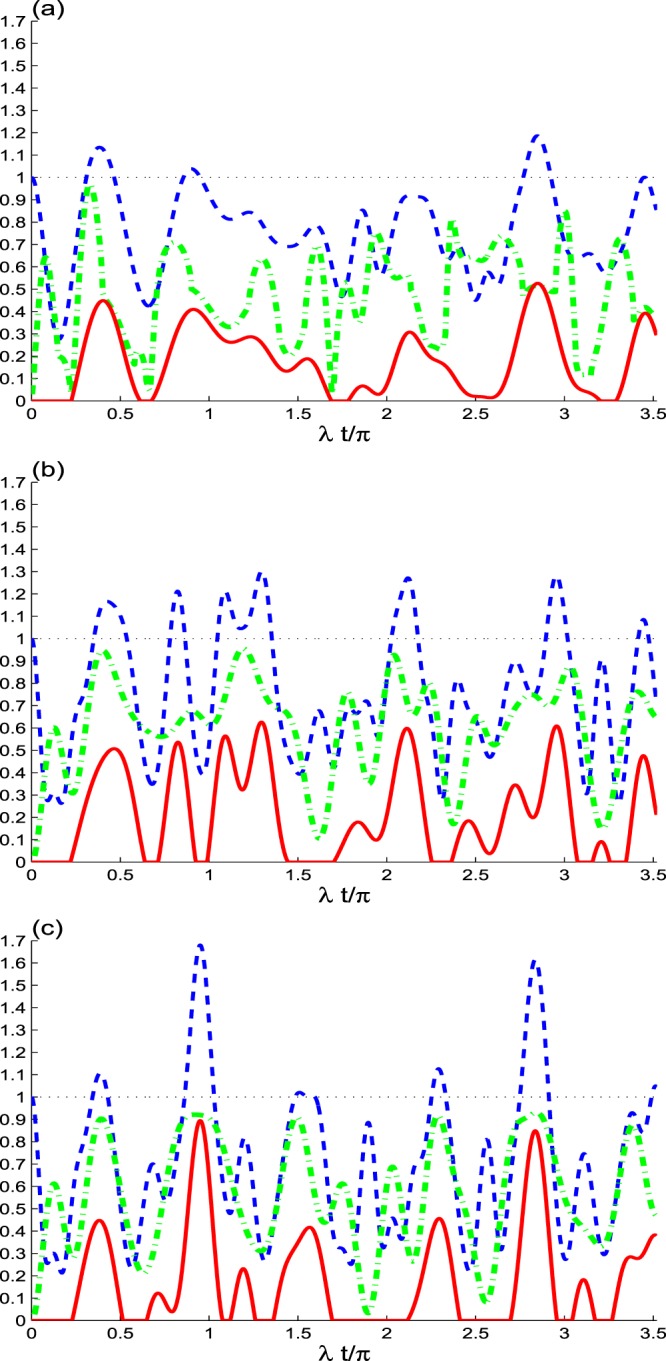
Time evolutions of *M*(*t*) (dashed dotted plots), *B*(*t*) (dashed plots) and *C*(*t*) (solid plots) with the dissipative rate *γ*/*λ* = 0 and *N* = 2 for different values of *κ* = 0 in (**a**), *κ* = 1 in (**b**) and *κ* = −1 in (**c**).

In Fig. 1, we display the resulted non-locality correlations of the two qubits with respect to the unitary interaction *γ* = 0. Where the trace-norm MIN, *M*(*t*), maximal Bell function, *B*(*t*), and the concurrence *C*(*t*) are displayed for different values of the superposition parameter *κ* = 0; *κ* = 0 in (a), *κ* = 1 in (b) and *κ* = −1 in (c) with the initial coherence intensity *N* = 2.

From a chosen initial pure state of the qubits |1_*A*_1_*B*_〉 or |0_*A*_0_*B*_〉, the elements of the correlation matrix R are zero except for *r*_33_ = 1, and the Bloch vector is *x* = (0, 0, ±1)^*t*^ ≠ 0. Therefore, *M*(0) = 0, *B*(0) = 1, and *C*(0) = 0, i.e, the state of the qubits does not have correlations. If the pure state of the qubits develops to one of the maximal correlated states, $\frac{1}{2}\mathrm{(|01}\rangle \pm \mathrm{|10}\rangle )$, the elements the correlation matrix *R* are zero except for the element *r*_11_ = ±0.5, *r*_22_ = ±0.5 and *r*_33_ = −0.5, and the Bloch vector is *x* = (0, 0, 0)^*t*^. Therefore, *M*(0) = 1, *B*(0) = 1.8284, and *C*(0) = 1 (generating maximal NLCs). Otherwise, the qubits-cavity interaction generates partial correlations.

In Fig. 1a, we consider the initial coherent state *κ* = 0 while the dipole decay of the qubits is neglected (*γ* = 0). We observe that the unitary interaction leads to: (1) the uncorrelated state of the qubits, |1_*A*_1_*B*_〉, produces an oscillatory partial entanglement and non-locality correlations during the time evolution of the qubits-cavity interaction. These partial correlations are enhanced with increasing the interaction time. As the unitary interaction evolves, the concurrence *C*(*t*) is zero for a short time, and it suddenly grows to its partial maximum value at particular points. These points are called growth-start points (GSPs). *C*(*t*) presents sudden birth and death entanglement^42^.

(2) the trace-norm MIN *M*(*t*) grows from zero (i.e., GSP is zero) to its partial maximum values. It has different behaviour compared to *C*(*t*). *M*(*t*) never vanishes. The upper bounds of trace-norm MIN are larger than that of the concurrence. (3) with respect to *B*(*t*), we observe that the Bell’s inequality is violated for short time intervals, in which *B*(*t*) > 1.

Where, in the case of *κ* = −1, the values of NLC functions may reach approximately the values of the maximal correlated states, $\frac{1}{2}\mathrm{(|01}\rangle \pm \mathrm{|10}\rangle )$, that are mentioned above. This mean that there is relation between the correlation dynamics and the evolution of the state of the qubits.

In Fig. 1b,c, the dependence of the NLCs on the superposition of coherent states parameter *κ* is depicted. The NLC functions for the two cases of the initial even coherent state *κ* = 1 in (b) and the initial odd coherent state *κ* = −1 in (c) are simulated with the same data of Fig. 1a.

We observe that the generated NLCs are bigger compared to the ones of the coherent cavity. The superposition of the coherent state parameter *κ* leads to the increase of the time intervals of the maximal violation of Bell’s inequality of *B*(*t*) > 1.

### Effect of the dipole qubits decay

**Figure 2:**
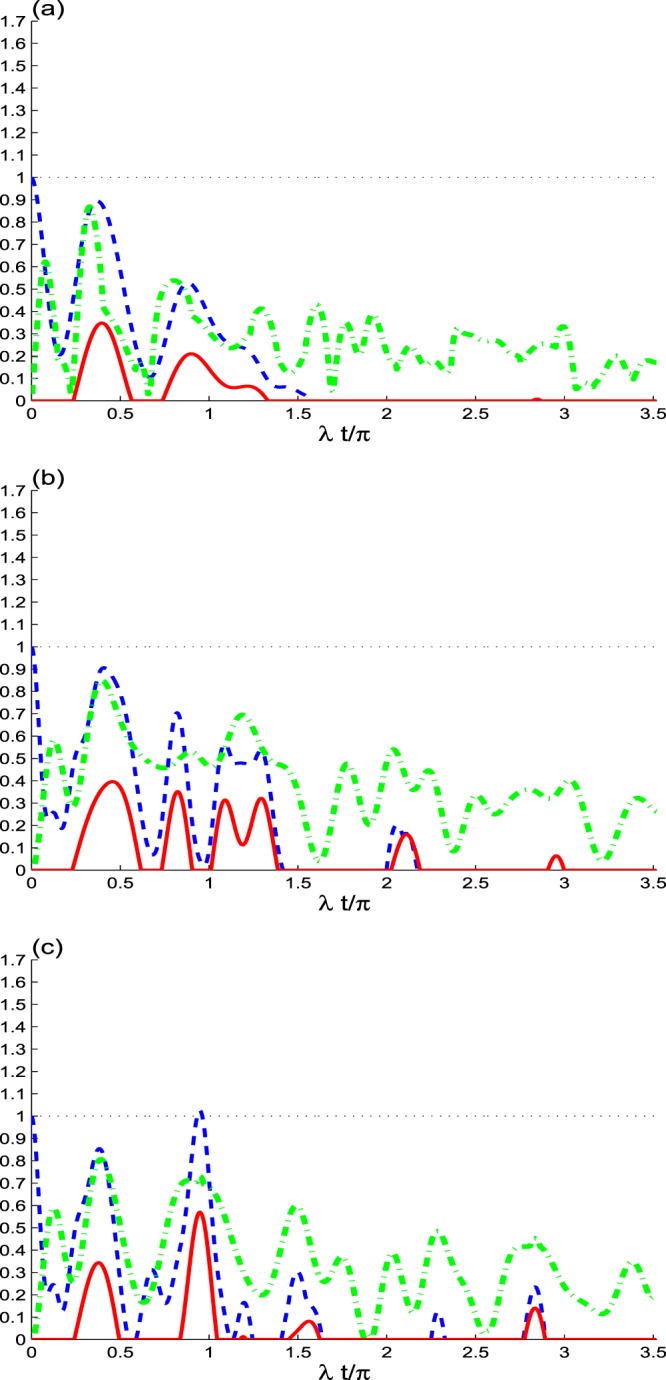
As Fig. 1, but with *γ* = 0.08*λ*.

In Fig. 2, the NLC functions *M*(*t*), *B*(*t*), and *C*(*t*) are plotted for the initial coherent state *κ* = 0 and the dipole decay of qubits (*γ* = 0.08*λ*). We note a more rapid deterioration of the non-local correlations. In case of coherent cavity field *κ* = 0 with *γ*/*λ* = 0.08, we observe: (1) due to the dipole decay of the qubits, the two functions of maximal Bell function and the concurrence vanish approximately whereas the trace norm MIN function does not vanish.

For the cases of the initial even coherent *κ* = 1 and odd coherent *κ* = −1 microcavity field, we observe that the oscillations, amplitudes and the negativity of the NLC functions *M*(*t*), *B*(*t*), and *C*(*t*) are more robust against the rate of dipole qubits decay *γ*/*λ*, (see Fig. 2b,c).

Therefore, the generated NLCs depend on the dipole decay and on the initial coherent cavity field. Due to the dipole qubits decay, the stable state of the two qubits has a value of the trace norm MIN correlation beyond that of entanglement and non-locality Bell-correlation.

### Effect of the initial coherence intensity

**Figure 3:**
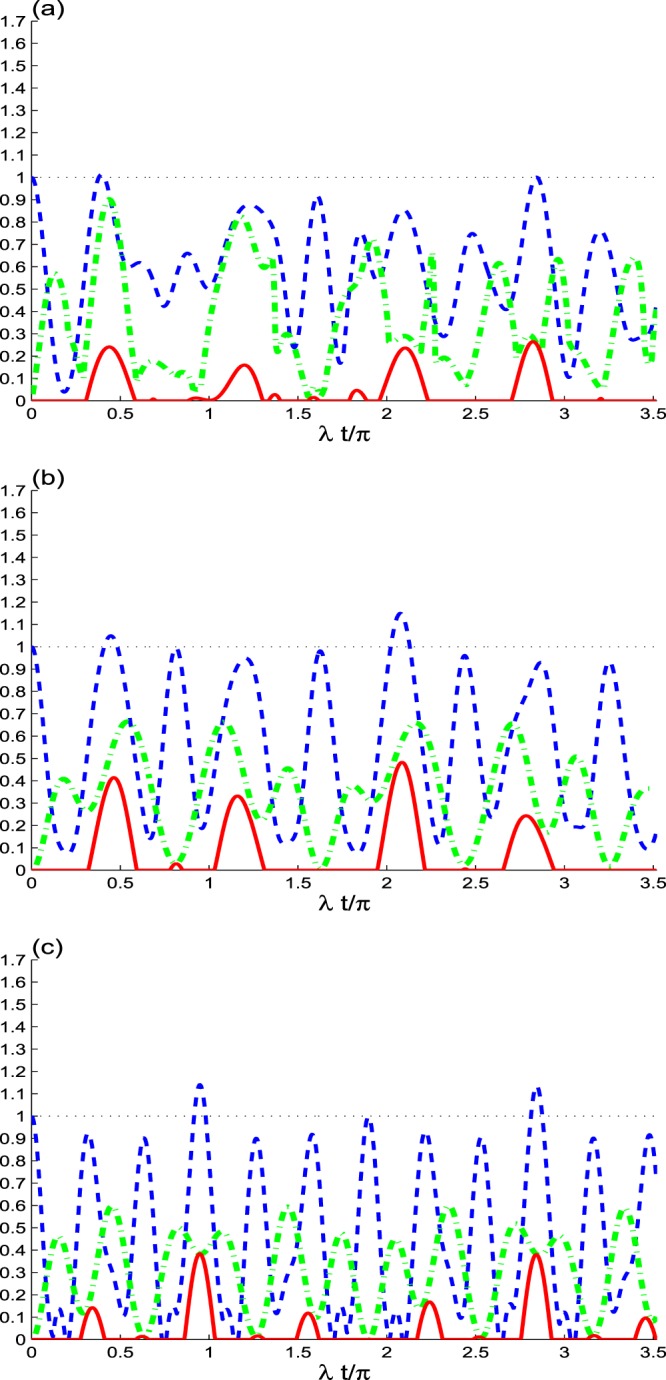
As Fig. 1(a), but with *α* = 0.5.

In Fig. 3, we analyze the effect of the initial coherence intensity *N*, where *N* = 0.5 is small and the dipole decay of qubits is neglected. We notice the change of the dynamical behavior of the NLCs. We observe that the NLC functions *M*(*t*), *B*(*t*), and *C*(*t*) (see Figs. 1 and 3) exhibit extreme values and amplitudes less than that of *N* = 2.

The Bell’s inequality is violated during short intervals, for the initial even coherent *κ* = 1 and odd coherent *κ* = −1 cavity field. However, for a coherent state there is no violation of the Bell’s inequality is observed. We deduce that the amount of the generated entanglement and non-locality correlations may be increased by increasing the initial coherence intensity *N*.

**Figure 4:**
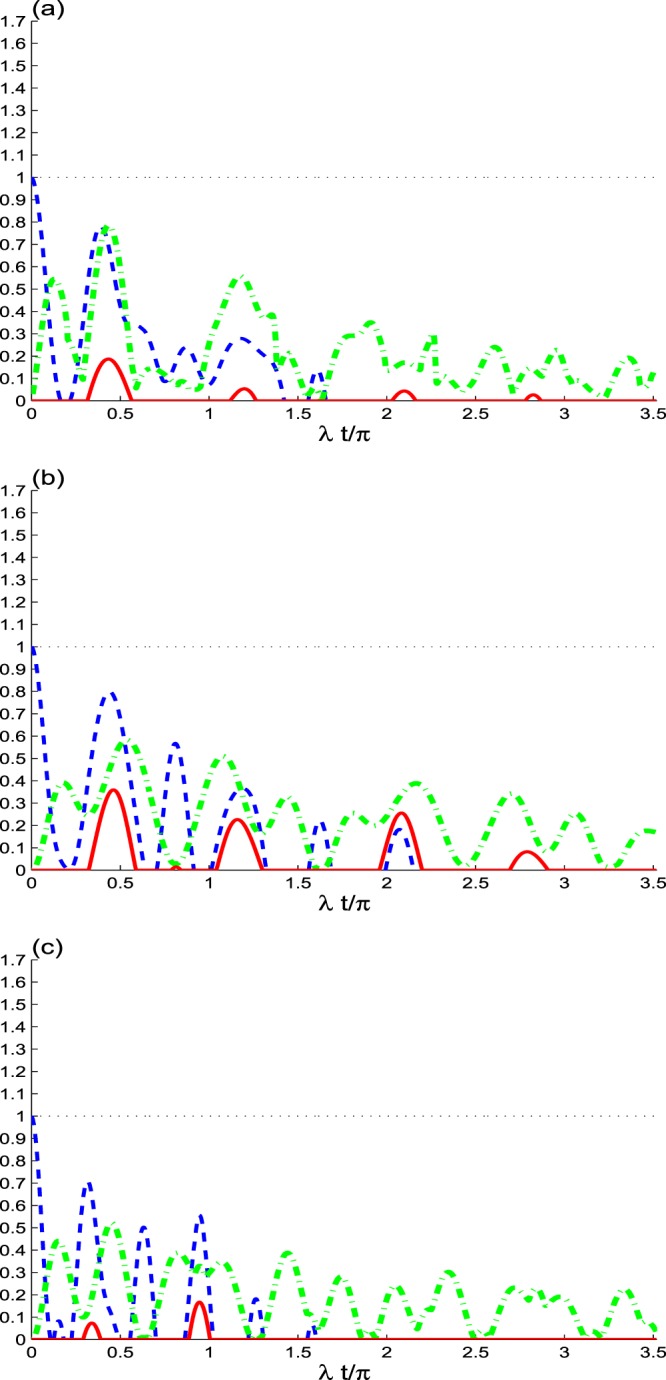
As Fig. 1, but for *α* = 0.5 with *γ* = 0.08*λ*.

Figure 4 shows the effect of the dipole decay *γ* = 0.08*λ* on NLCs between the two qubits. We observe that the NLCs for *γ* = 0.05*λ* have damped oscillations, their amplitudes decrease until completely vanish. When the dipole decay is increased, reduction of the final disappearance time of the NCLs is noted. For the small initial coherence intensity, *N* = 0.5 (see Fig. 4b), *C*(*t*) is quasi-periodic. The sudden birth and death entanglement is observed for large time windows. The generated NLCs are weak and has less robustness as the initial coherence intensity decreases. The NLCs are reduced by the decrease of the initial coherence. The extreme values of trace norm MIN is more robust than the entanglement and non-locality Bell-correlation.

## Conclusion

Throughout this paper, an analytical description of a cavity contains two qubits spatially separated is established. The non-locality correlations [including trace norm measurement induced non-locality, maximal Bell-correlation and concurrence entanglement] of the two qubits are explored via the trace norm measurement induced non-locality and the Maximum Bell function. The rise in two-qubit damping rates induces a fast deterioration of the coherence. We notice that this system presents sudden birth and death entanglement. The generated non-locality correlations essentially determined by the loss rate of the two-qubit and the initial coherence of the cavity.
